# Environment Understanding Algorithm for Substation Inspection Robot Based on Improved DeepLab V3+

**DOI:** 10.3390/jimaging8100257

**Published:** 2022-09-21

**Authors:** Ping Wang, Chuanxue Li, Qiang Yang, Lin Fu, Fan Yu, Lixiao Min, Dequan Guo, Xinming Li

**Affiliations:** 1 School of Network & Communication Engineering, Chengdu Technological University, Chengdu 610031, China; 2 School of Automation, Chengdu University of Information Technology, Chengdu 610225, China; 3 School of Computer & Network Security (Oxford Brooke College), Chengdu University of Technology, Chengdu 610059, China

**Keywords:** environment understanding algorithm, substation inspection robot, DeepLab V3+, ASPP, CBAM

## Abstract

Compared with traditional manual inspection, inspection robots can not only meet the all-weather, real-time, and accurate inspection needs of substation inspection, they also reduce the work intensity of operation and maintenance personnel and decrease the probability of safety accidents. For the urgent demand of substation inspection robot intelligence enhancement, an environment understanding algorithm is proposed in this paper, which is an improved DeepLab V3+ neural network. The improved neural network replaces the original dilate rate combination in the ASPP (atrous spatial pyramid pooling) module with a new dilate rate combination with better segmentation accuracy of object edges and adds a CBAM (convolutional block attention module) in the two up-samplings, respectively. In order to be transplanted to the embedded platform with limited computing resources, the improved neural network is compressed. Multiple sets of comparative experiments on the standard dataset PASCAL VOC 2012 and the substation dataset have been made. Experimental results show that, compared with the DeepLab V3+, the improved DeepLab V3+ has a mean intersection-over-union (mIoU) of eight categories of 57.65% on the substation dataset, with an improvement of 6.39%, and the model size of 13.9 M, with a decrease of 147.1 M.

## 1. Introduction

The substation, one of the important components of the power grid, is also the basis for measuring and controlling the power grid [1,2] and is responsible for the regulation of voltage rise and fall and the distribution of electrical energy in the power network. In order to ensure the normal operation of the substation, it is necessary to conduct regular inspection of the operation state of the power equipment in the substation, so as to eliminate the power security risks in an early and timely manner [3,4,5]. With the deepening of smart grid construction [6], inspection robots have been widely used in substations, which are mainly used to replace manual operations in substation inspection, such as emergency, difficulty, low efficiency, and low intelligence [7,8], and have gradually achieved good effect. Now, it is one of the important research fields [9,10,11]. In terms of intelligent inspection robots, the basic premise of their complex inspection tasks is whether they can effectively understand their inspection road environment.

Environment understanding, as the name implies, means that the inspection robot captures information about the robot’s surroundings using its own configured sensors and effectively preprocesses and fuses this data through relevant algorithms to construct a mathematical model of the deep semantic features of the environment [12]. Finally, it generates a graph from sensor data to represent the relationship between the detected objects [13,14]. Its own configured sensors are mainly ultrasonic radar sensors, LiDAR sensors, and visible light sensors. Different sensors have different ways of collecting environmental information, and the collected data are also different. Thus, according to the type of sensors, the robot’s approach to environment understanding can also be divided into: ultrasonic radar-based environment understanding, LiDAR-based environment understanding, visible light sensor-based environment understanding [15], etc.

With the development of deep learning technology, vision-based environment understanding methods have gradually become a hot spot and focus of environment understanding research. At present, deep learning-based environment understanding algorithms are mainly divided into two categories. One is the target detection method, which can detect the target object category and coordinates in the image. The other is semantic segmentation. The semantic segmentation method improves the target location to the pixel level, namely classifying each pixel in the image point by point. 

The rapid development of deep learning techniques has breathed new life into the research of semantic segmentation. The landmark event was the first semantic segmentation neural network-FCN (fully convolutional network) proposed by Long et al. in 2014, which features a VGG-16 network based on the use of convolutional layers, instead of fully connected layers, and uses cross-layer as well as bilinear interpolation to transform the segmentation results from coarse to fine [16]. However, FCN is less accurate at predicting small targets and loses a lot of features in the down-sampling stage. Therefore, in recent years, a large number of convolutional neural networks have emerged for image semantic segmentation, such as PSPNet, SegNet, DANet, CCNet, OCNet, ACNet, and so on. In 2016, Zhao et al proposed a pyramid scene parsing network (PSPNet) by using a pyramid pooling module to aggregate the context information of different regions, which is able to capture global information better than other networks [17]. In 2017, Badrinarayanan et al proposed a semantic segmentation network based on decoders and encoders, namely SegNet network, which is characterized by converting maximum pooling indexes to encoders [18]. In 2019, Fu et al. proposed the DANet network, which attaches a position attention module (PAM) and a channel attention module (CAM) to the expanded FCN [19]. The network can enrich the context information and improve the segmentation accuracy of the model. In order to obtain the contextual information of the whole image more effectively and efficiently, Huang et al proposed CCNet, which uses a novel cross-attention module for each pixel to collect contextual information about the pixel [20]. Similar to CCNet, OCNet [21] uses non-native modules to collect contextual information. It can enhance the role of object information and solve semantic segmentation problems. In addition, for the purpose of enhancing feature extraction without increasing the amount of computation, ACNet [22,23] was proposed. The network can intensify feature extraction during the training phase and focus on the convolutional nucleation ensemble during the testing phase. As a result, more high-quality features from different channels can be taken advantage of, without adding any amount of computation.

Furthermore, up to now, a large number of converter-based methods for image semantic segmentation have emerged, like SegFormer, MaskFormer, Trans4Trans, and SETR. In 2021, Xie et al. proposed a simple, efficient, and powerful semantic segmentation algorithm, SegFormer, which combines a transformer with lightweight multilayer perceptrons that not only require position encoding, but also avoid complex decoders [24]. In the algorithm, MLP decoders aggregate information from different layers so that both local and global attention are powerfully expressive. In the same year, Cheng et al proposed the MaskFormer approach, which can convert any existing per-pixel classification model into a mask classification [25]. Then, Zhang et al. proposed Trans4Trans, which establishes transformer-based encoders and decoders to take full advantage of the contextual modeling capabilities of the self-attention layer in remote transformers [26]. Moreover, Zheng et al proposed SETR, which replaces convolutional layer-based encoders with a pure transformer to gradually reduce the spatial resolution, resulting in a new segmentation model that makes it more efficient to select high-quality features [27].

To solve the problems of lack of spatial consistency, lack of details, and not considering inter-pixel relationships in FCN, Chen et al proposed DeepLab network in 2014, which adds fully connected, conditional random fields at the end of FCN and performs boundary optimization on the output results of FCN [28]. In recent years, due to more and more researchers’ attention, the DeepLab network [29,30,31,32] has overcome the loss of details caused by the repeated max pooling and down-sampling in the deep convolutional neural network, resolution reduction caused by continuous pooling or convolution, and the obstruction of spatial invariance. Finally, the DeepLab network achieves semantically precise pixel location and high accuracy of segmentation boundaries.

By analyzing and comparing the current mainstream deep learning semantic segmentation networks, an improved DeepLab V3+ semantic segmentation network for substation environment understanding is proposed in this paper, and the contributions are summarized as follows.

(1) The improved algorithm uses the new dilate rate combination (2, 4, 6, 8) to replace the original dilate rate combination, which increases the accuracy of the network for low-resolution feature extraction and has better segmentation accuracy of object edges;
(2) CBAM is added to the two up-samplings, respectively. A decoder based on CBAM in the improved DeepLab V3+ network can effectively improve the utilization of image pixel information;
(3) Network size is compressed. The size of the proposed environment understanding algorithm model in this paper is 13.9 M, with a decrease of 147.1 M, which benefits transplantation to the embedded platform with limited computing resources.

The paper is organized as follows: The development process of DeepLab and how to improve the DeepLab algorithm are presented in Section 2. Experimental results and analysis are discussed in Section 3. Finally, the conclusion is described in Section 4.

## 2. Improvement Strategies for DeepLab Networks

This section first briefly introduces the development process of DeepLab in Section 2.1, and then introduces the network structure of the improved DeepLabV3+ in Section 2.2.

### 2.1. Development of DeepLab

**Figure 1:**
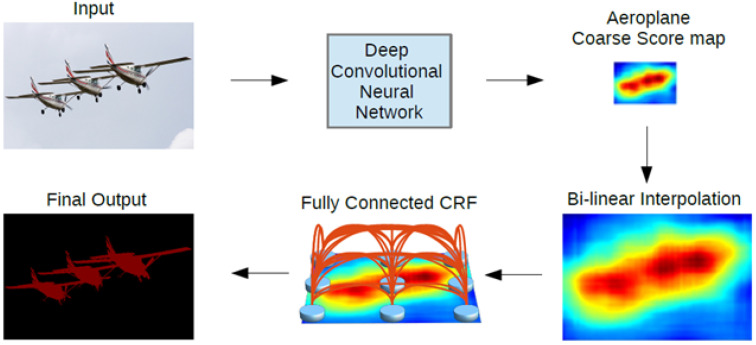
DeepLab V1 network structure.

After continuous efforts, the Google team proposed the DeepLab series of networks. In order to solve the inability to precisely locate the semantics of pixel points, DeepLab V1 [28] combined the ideas of both deep convolutional neural networks and fully connected conditional random fields and used VGG16 as the backbone network for feature extraction; it also added the last layer to the fully connected, conditional random fields (CRF), which can produce semantically accurate predictions and detailed segmentation maps with high computational efficiency. Meanwhile, the problem of decreasing resolution brought by repetitive maximum pooling and down-sampling in deep convolutional neural networks, where the decreasing resolution loses details, DeepLab V1 used convolutional layers with dilate rate to extend the perceptual field to obtain more contextual semantic information. Its network structure is shown in Figure 1.

**Figure 2:**
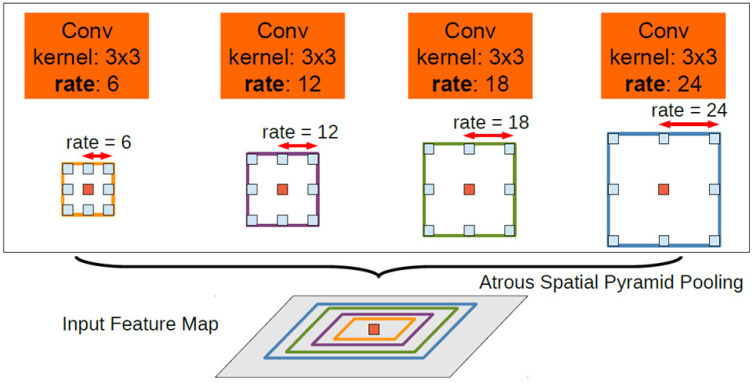
ASPP network structure [29].

Compared with DeepLab V1, DeepLab V2 [29] still maintained the process of Figure 1, but made three improvements on the basis of V1, which were the dilate rate algorithm, replacement of feature extraction network (replace VGG16 with ResNet), and proposed ASPP. These improvements advanced the semantic segmentation effect of the model. The network structure of ASPP is shown in Figure 2.

On the basis of DeepLab V2, in order to solve the resolution decline caused by continuous pooling or convolution and the obstruction of spatial invariance to the segmentation task, DeepLab V3 [30] also made three improvements, which are the usage of deeper atrous convolution, the addition of batch normalization (BN) after the ASPP, and the conversion of the large sampling rate of the 3 × 3 atrous convolution to 1 × 1 convolution, in order to capture more global semantic information.

**Figure 3:**
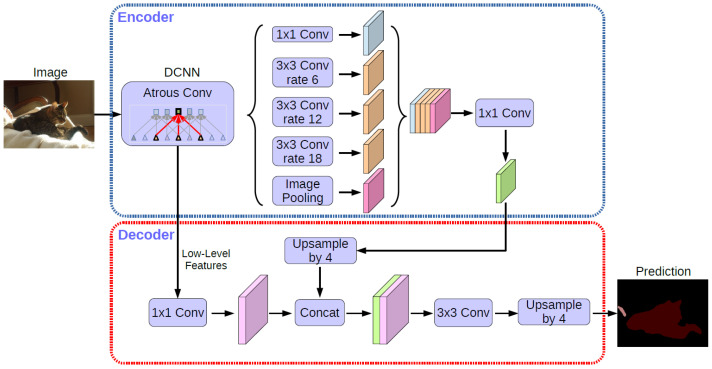
DeepLab V3+ network structure.

DeepLab V3+ [31] mainly fused the ASPP and encoder–decoder structure. The main body of the encoder is a deep, convolutional neural network with atrous convolution, which can be used in common classification networks, such as ResNet and Xception [32]. The ASPP module contains atrous convolution, which is used for introducing multi-scale information. Compared with DeepLab V3, DeepLab V3+ introduced the decoder module, which further integrated low-level features and high-level features to improve the accuracy of the segmentation boundary. In a sense, DeepLab V3+ leads into the idea of encoder–decoder on the basis of Dilated-FCN. The network structure is shown in Figure 3. The semantic segmentation steps of DeepLab V3+ are described as follows:

1. Input the image to be tested;
2. Use the deep convolutional neural networks (DCNN) for different levels of convolution, which probes convolutional features at multiple scales by applying atrous convolution with different rates, with the image-level features.
3. The encoder features from 1 × 1 convolution are up-sampled by a factor of 4 and then concatenated with the corresponding low-level features from the network backbone that have the same spatial resolution.
4. After the concatenation, a few 3 × 3 convolutions are used to refine the features, followed by another simple bilinear up-sampling by a factor of 4 to output prediction results.

Although the DeepLab V3+ network is the most advanced one in DeepLab series, it still has the following problems: first, as far as the algorithmic model is concerned, the main shortcomings are that the dilate rate combination method of the ASPP module is not suitable for the segmentation of target edge contour, the amplitude of up-sampling is too large to lose semantic features, and the number of layers in the network is large, and most of them are ordinary convolution, resulting in a large amount of computation. Second, in terms of the prediction map results, without target edge segmentation accuracy, the mIoU of its road recognition accuracy is only 77.07%, which fails to meet the actual engineering requirements, and the model size of 168 MB is not easy to embed into the inspection robot system. Therefore, in order to improve the shortages of the network, the improved DeepLab V3+ network is proposed in this paper.

### 2.2. Improved DeepLab V3+ Network Structure

**Figure 4:**
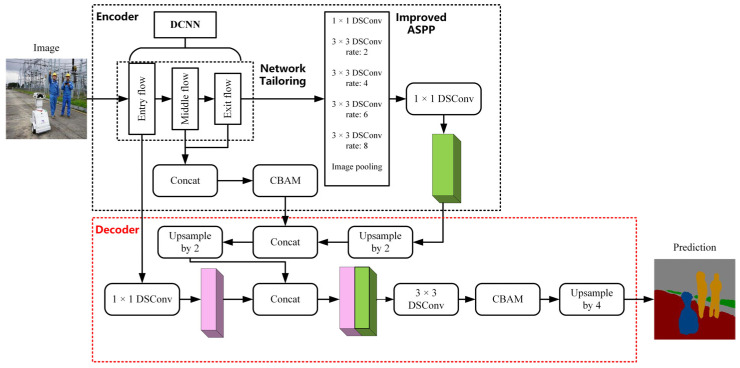
Improved DeepLab V3+ network structure.

The network structure is mainly divided into two parts, the encoding part and the decoding part. The improved network structure is shown in Figure 4.

**Figure 5:**
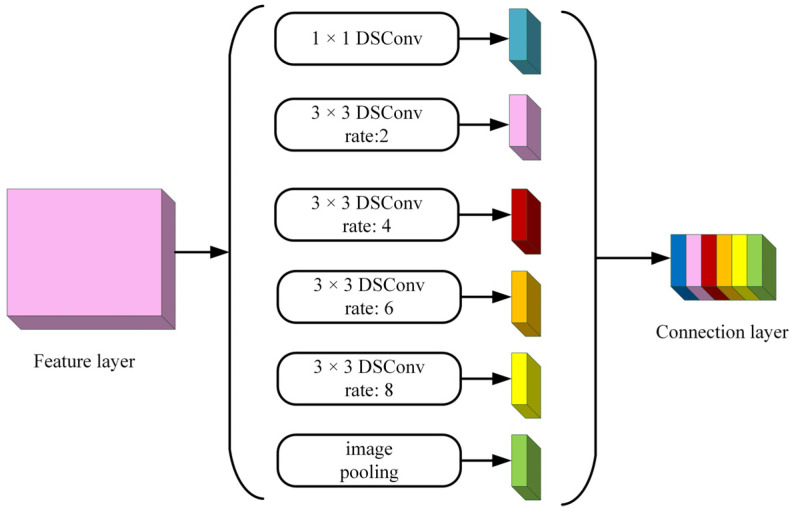
ASPP structure.

In the encoding part, it mainly contains DCNN, the ASPP module, feature fusion of channel directions, and deep separable convolution, discarding the labeled convolution method and adding one feature fusion operation inside the deep convolutional neural network. Aiming at the problem that the existing dilation rate combination makes the up-sampling range large and loses the segmentation feature information, on the basis of experimental results, the network replaces the original combination of (6, 12, 18) with the new combination of (2, 4, 6, 8) to improve the segmentation accuracy of low-resolution targets and their edges in the ASPP module. The improved ASPP structure is shown in Figure 5.

In the decoding part, the operations of up-sampling, feature fusion, and convolution are mainly included, and all the ordinary convolution is replaced by depth-separable convolution to reduce the model calculation. In particular, the improved structure adds a feature fusion operation. In the stage of feature extraction network, the resolution of the image is reduced by 16 times, and the semantic features of each part are extremely important for the final results. The original network only uses the semantic information of the low levels and high levels in the feature extraction network. Therefore, this paper adds a feature fusion operation between the middle-level and high-level semantic features in the feature extraction network. Later, the CBAM [33] is introduced to handle the problem of low target edge segmentation accuracy.

**Figure 6:**
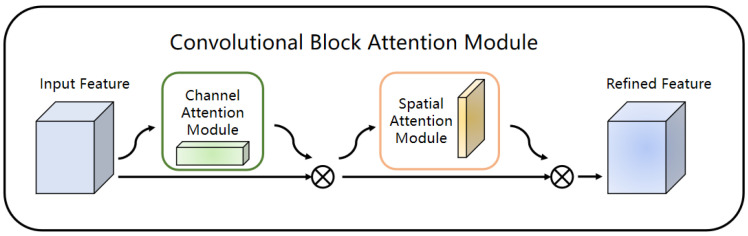
CBAM structure.

CBAM is a module that combines spatial and channel attention mechanisms to obtain the target area that needs to be focused on for obtaining more details and key information of the current task in image semantic segmentation. It can improve the accuracy of segmentation results, and has better interpretability and generality. Its structure is shown in Figure 6. 

CBAM can amplify the weight of the effective feature channels in the feature layer so that the model can better distinguish the target and the background. Therefore, it can solve the problem of the outdoor obstacle features of the substation being difficult to extract under complex background conditions. By applying spatial attention and channel attention, CBAM can enhance the semantic information of the outdoor obstacle features of the substation and suppress the interference of complex background information; as a result, the network can focus on the feature information of obstacle targets in the process of generating the feature layer. In this way, the problem of poor recognition accuracy of obstacles under complex background and changing lighting conditions from unstructured outdoor environments can be effectively alleviated. Therefore, this paper considers introducing CBAM into the DeepLab V3+ to improve the anti-environmental interference ability of the model.

In order to cope with the pixel information loss caused by the direct up-sampling by a factor of 4 of the original network, the continuity of semantic information in the reduction process is increased by two small-scale up-samplings. For the first time, the feature map after ASPP processing and channel compression in the encoding part is double up-sampled, and then the feature fusion is carried out with the feature information obtained by CBAM in the encoding part. Finally, the result of feature fusion is double up-sampled. The subsequent operation is feature fusion, with the low-level semantic features of Xception, followed by a 3 × 3 depth-separable convolution. In particular, the improved network adds a CBAM before the second up-sampling by a factor of 4.

The specific embedded system for this paper is Raspberry Pi 3B+, with only 1 G of memory. The substation inspection robot described in this paper undertakes tasks from many application scenarios, such as intelligent video analysis, multi-sensor information fusion (ultrasonic radar, odometer, visible light camera), infrared identification of power equipment, identification of environmental obstacles in substations, identification of power equipment defects, robot navigation algorithm (3D LiDAR visual SLAM), environmental status monitoring (temperature and humidity monitoring, meter recognition), partial discharge detection, network communication, etc. If the model size of this paper reaches 177.00 MB, the specific embedded system cannot meet the needs of the above-mentioned application scenarios. 

Restricted by the limited computing resources of the embedded platform of the substation inspection robot, the size of the improved DeepLab V3+ network must be less than 20 M to meet the embedded platform requirements. Therefore, it is necessary to prune the size of the improved network. 

**Figure 7:**
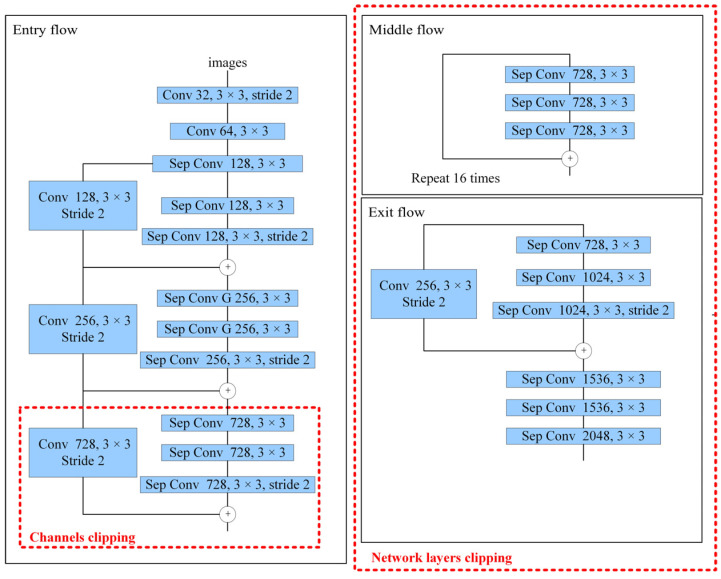
Xception clip.

Based on the above analysis, in order to meet the needs of embedded platform transplantation, this paper clips the Xception feature extraction network to reduce the size of the model. Model clipping consists of two parts: network layers clipping and channels clipping, which are shown in Figure 7. First, in terms of network layers clipping, since the features extracted at the end of the network are more abstract and have less detail and edge information, this paper removes middle flow and exit flow at the end of Xception. Second, in terms of network channels clipping, in order to remove redundant information brought by multiple channels and preserve feature information as much as possible, this paper changes the number of channels of the three convolutional layers at the end of the remaining entry flow from the 728 to 512. After the above two steps, the model size is compressed from 177.00 M to 13.90 M. Experiments show that the improved DeepLab V3+ after clipping has less than 1% loss of accuracy, as compared with before. Therefore, in regards to model size and recognition accuracy, it can meet the deployment requirements of the embedded platform in the substation inspection robot.

Finally, a list of common embedded systems is shown in the Table 1. In the last two columns of the table, “Yes” means the network can be used in the embedded system, and “No” means the network cannot be used in the embedded system.

## 3. Experimental Results and Analysis

This section will elaborate from three aspects: experimental dataset, evaluation index, and result analysis.

### 3.1. Experimental Dataset

The original image acquisition was mainly through the visible light sensor carried by the substation inspection robot to obtain the video of the substation inspection scene and convert the acquired video to image for labeling. The acquisition locations were distributed in several different substations; the acquisition time covers three periods: morning, noon, and afternoon. Acquisition scenarios cover different weather conditions (sunny, cloudy, rainy, etc.), and then data pre-processing (video to image, de-duplication, de-blurring, resolution normalization) was performed on the acquired videos. Finally, Labelme software was used for labeling.

**Figure 8:**
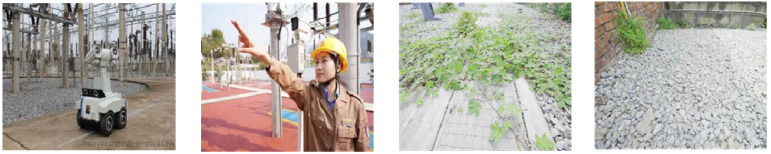
Main obstacle types.

Finally, 4055 images were collected in the substation dataset, and the target categories contained eight categories: background, road, weeds, rocks, pedestrians, robots, fences, and road pits. The main obstacles were robots, pedestrians, weeds, and rocks, which are shown in Figure 8. The class distribution of the dataset is: 4055 images including background, 3906 images including road, 3855 images, including weeds, 941 images including rocks, 253 images of robots, 226 images including fences, 217 images including pedestrians, and 68 images including road pits. In the dataset, 3244 images were randomly selected as the training set, 405 as the validation set, and the remaining 406 as the test set.

The hardware of model training was a desktop computer equipped with NVIDIA’s eForce RTX 3090, and the deep learning framework was Keras (based on TensorFlow). The basic parameters of the network training in this paper are as follows: learning rate was 1 × 10^−4^; batch size was 4; learning optimizer was Adam (lr = lr); loss function was ‘categorical_crossentropy’.

### 3.2. Evaluation Criteria

In this paper, the evaluation indexes of network accuracy were the mIoU and the mean average precision (mAP), which are introduced as follows.

(1) *mIoU*

The mIoU is the sum-average result of the intersection-over-union for each type, which is the ratio of the intersection and merge of the actual and predicted category samples; the formula is as follows:

(1) $$mIoU=\frac{1}{k+1}\sum_{0}^{k}\frac{TP}{FN+FP+TP}$$

where, *k* is the number of categories; *TP*, *FP*, and *FN* represent a true positive, false positive, and false negative, respectively.

(2) *mAP*

The formula is shown in (2):

(2) $$mAP=\int_{0}^{1}P(R)dR$$

where, *R* denotes the recall rate and *P* denotes the accuracy rate.

### 3.3. Analysis of Experimental Results

On the basis of the DeepLab V3+ structure, this article replaced the original dilate rate combination in the ASPP module with a new dilate rate combination and added a CBAM in the two up-samplings, respectively. In order to meet the demands of the specific embedded platform with limited computing resources, the proposed neural network was clipped. After the three networks (DeepLab V3+, improved DeepLab V3+, and improved DeepLab V3+(clipping)) were independently trained on the standard dataset PASCAL VOC 2012 and the substation dataset, the performance of the three networks for image semantic segmentation was compared under the same testing set. Experimental results showed that the improved DeepLab V3+ is better than the DeepLab V3+, with respect to the mIoU, and improved DeepLab V3+(clipping) is better than the improved DeepLab V3+, in regards to model size, with less than 1% loss of accuracy.

(1) ASPP module optimal dilate rate combination experiment

The size of the dilate rate of the atrous convolution can expand or reduce the receptive field of the feature map without adding additional computational effort [34]. The size of the receptive field of the feature map directly affects the recognition accuracy of targets of different sizes. Therefore, it is extremely important to find the optimal combination of the dilate rate. Due to the feature extraction network of DeepLab V3+, the network reduces the image resolution by 16 times, and the substation inspection scene is dominated by small targets. Therefore, this paper chose more combinations of smaller dilate rate. For this reason, different combinations of dilate rate were selected for the ASPP module of DeepLab V3+ network in this paper, and experiments were conducted on the standard dataset PASCAL VOC 2012 and the substation dataset, respectively.

As can be seen from Table 2, the dilate rate combination (2, 4, 6, 8) of ASPP module both had the best mIoU on the standard dataset PASCAL VOC 2012 and the substation dataset. Therefore, in this paper, the dilate rate combination (2, 4, 6, 8) was chosen as the dilate rate combination for the ASPP module in the improved network.

(2) Exploring the optimal position of CBAM and the new way of up-sampling experiment

Based on the DeepLab V3+ network, this paper improves the way of up-sampling. In order to further improve the network accuracy of the sampling part of the network, CBAM is introduced in this paper. When introducing the CBAM, its position in the network needs to be determined. In this paper, three positions are explored, including after the fusion of middle- and high-level semantic features of the feature extraction network, before the second up-sampling by a factor of 4, and the combination of the first two approaches. A comparative experiment with the network without CBAM and the network under comprehensive improvement has been made. The experimental results are shown in Table 3. 

From Table 3, taking no account of the influence of ASPP, it can be seen that the model performance was optimal when CBAM was introduced into the two up-sampling. The mIoU in PASCAL VOC 2012 dataset reached 79.34%, and that in the substation dataset reached 55.64%. Therefore, in this paper, the improvement network was chosen to introduce the CBAM for both up-samplings as the improvement scheme. In addition, further joint experiments were done in this paper; that is, the joint ASPP module optimal dilate rate combination and the CBAM optimal location were improved in two ways, and their mIoU were 79.81% in the PASCAL VOC 2012 dataset and 57.82% in the substation dataset. The joint experiment showed that the improvements made in this paper are effective and have an enhancement effect on the accuracy of the network.

(3) Model clipping experiments

In order to make the substation environment understanding network be transplanted into the embedded platform of the substation inspection robot, this paper compressed the network and conducted experiments on the substation dataset. The experimental results are shown in Table 4. 

As shown in Table 4, the mIoU of the improved DeepLab V3+ is 57.83%, which is better than that of the DeepLab V3+. In addition, the parameter size of the improved DeepLab V3+ (clipping) is only 13.9 M, which is much lower than that of the DeepLab V3+. The accuracy difference of mIoU is only 0.18% between the improved DeepLab V3+ and the improved DeepLab V3+ (clipping). As is known to all, FLOPs refer to the theoretical calculation amount, which is used to measure the complexity of the algorithm or model, and is related to the speed of the algorithm. As can be seen from Table 4, the theoretical calculation amount of the improved DeepLab V3+ (clipping) in this paper is only 1.32% of the DeepLab V3+. It indicates that the proposed approach is better than the DeepLab V3+, with respect to model complexity and algorithm speed. Therefore, considering the three indicators (parameter, FLOPS, and mIoU), the improved DeepLab V3+ (clipping) is the most suitable to be transplanted to the embedded platform in the inspection robot.

Furthermore, eight categories of the improved model in the substation dataset were tested in this paper, and the main categories tested were robots, pedestrians, weeds, and rocks. It is worth mentioning that the evaluation index of the test is IoU, and the results of the test are shown in Table 5. 

From Table 5, the mIoU of the seven categories has increased to some extent. In the seven categories, the increase of the mIoU of ROAD is the largest, and the increase of the mIoU of ROAD PITS is the smallest. The reason is that on the one hand, as the introduction of CBAM increases the segmentation accuracy of image edges, the proposed network can more accurately distinguish roads and other objects; on the other hand, due to the color similarity between ROAD PITS and ROAD, it is difficult for researchers to mark the ROAD PITS. According to the data in Table 5, the mIoU of eight categories by the improved DeepLab V3+(clipping) network is 57.65%, with an improvement of 6.39%.

**Figure 9:**
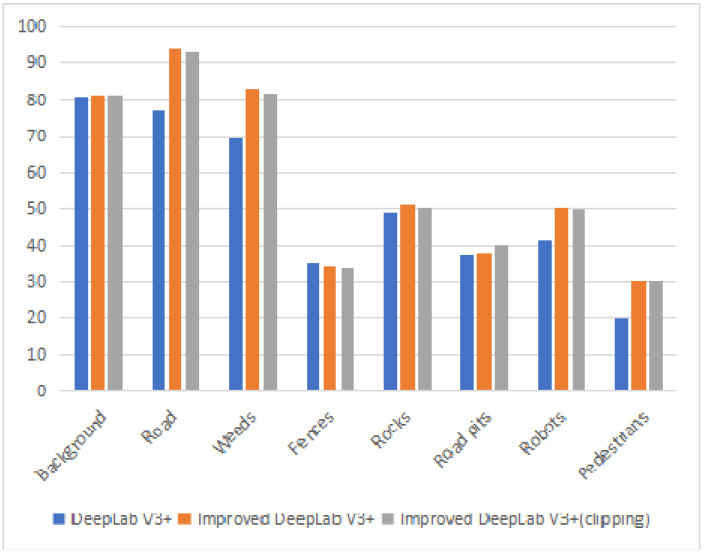
IoU test results for 8 types of targets in substations.

Figure 9 shows that the improved DeepLab V3+ has better experimental results than DeepLab V3 among seven target categories. Moreover, the IoU of the main obstacles are enhanced, and the minimum enhancement is 1.49%. In addition, the IoUs of weeds, rocks, robots, and pedestrians have an improvement of 12.94%, 2.54%, 9.17%, and 10.53%, respectively. 

**Figure 10:**
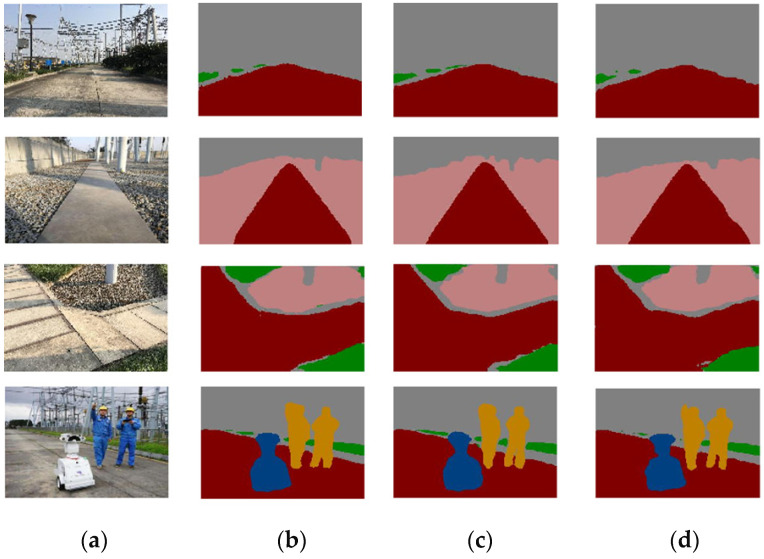
Network detection effect before and after improvement: (**a**) original diagram of substation; (**b**) DeepLab V3+; (**c**) improved DeepLab V3+; (**d**) improved DeepLab V3+(clipping).

The effectiveness of the improved network in this paper was verified by experiments, and the comparison results before and after the network improvement are shown in Figure 10.

## 4. Conclusions

This paper proposes an improved network based on the DeepLab V3+ network. Based on this network, an intelligent inspection robot can be better applied to substation inspection. In this paper, a new dilate rate combination (2, 4, 6, 8) in ASPP is proposed, which has the best mIoU in experiments. Meanwhile, the CBAM is introduced. Based on the validation of different datasets, the results show that, compared with the DeepLab V3+ network, the improved DeepLab V3+ network has higher network accuracy and network efficiency. Experimental results indicate the effectiveness of the introduced method for substation inspection. Our future work will deeply explore the inner law of the network feature fusion method to improve the recognition accuracy; in addition, greater progress should be made in the balance of network accuracy and network efficiency. An example would be, expanding the capacity of the dataset and improving the inference speed in order to improve the inspection efficiency of substations to a greater extent.

## Figures and Tables

**Table 1:** A list of common embedded systems.

No.	Common Embedded Systems	Memory	Before	After
1	STM32-F407	16 MB	No	No
2	FPGA-Mini (EP4CE10)	34 MB	No	Yes
3	FPGA-Pro (XC6SLX16)	144 MB	No	Yes
4	Raspberry Pi 3B+	1 GB	Yes	Yes
5	NVIDIA Jetson TX2	4 GB	Yes	Yes

**Table 2:** Experimental results of mAP and mIoU corresponding to different cavitation rates.

Dilate Rate Combination	PASCAL VOC 2012		Substation Dataset	
	mIoU (%)	mAP (%)	mIoU (%)	mAP (%)
(6, 12, 18)	78.85	77.31	51.25	70.40
(6, 12, 18, 24)	78.92	77.50	51.95	70.60
(4, 8, 12, 16)	79.09	78.63	52.73	72.35
(2, 4, 6, 8)	79.18	78.95	54.63	74.49

**Table 3:** MAP and mIoU test results by network.

Network Structure	PASCAL VOC 2012		Substation Dataset	
	mIoU (%)	mAP (%)	mIoU (%)	mAP (%)
DCNN	78.85	77.31	51.25	70.4
DCNN + CBAM	79.01	78.14	54.76	70.2
DCNN (+CBAM)	78.14	77.05	52.19	71.12
DCNN (+CBAM) + CBAM	79.34	78.62	55.64	71.73
DCNN (+CBAM) + CBAM + ASPP *	79.81	79.11	57.82	72.91

Note: ASPP * is the improved ASPP module.

**Table 4:** MAP and mIoU test results by different network.

Network Name	Substation Dataset			
	Parameter (MB)	FLOPs (10^6^)	mIoU (%)	mAP (%)
DeepLab V3+	161.00	42.50	51.26	74.40
Improved DeepLab V3+	177.00	46.10	57.83	72.90
Improved DeepLab V3+(clipping)	13.90	0.56	57.65	73.10

**Table 5:** IoU test results of each category.

Category	Substation Dataset (%)		
	DeepLab V3+	Improved DeepLab V3+	Improved DeepLab V3 + (Clipping)
Background	80.32	81.33	81.30
Road	77.07	94.11	93.29
Weeds	69.97	82.91	81.87
Fences	34.98	33.95	33.80
Rocks	48.95	51.49	50.44
Road pits	37.74	38.13	39.97
Robots	41.26	50.43	50.10
Pedestrians	19.75	30.28	30.41
mIoU	51.26	57.83	57.65

## Data Availability

The data used to support the findings of this study are available from the corresponding author upon request.
